# Third Renal Transplant in a Patient With Prune Belly Syndrome

**DOI:** 10.7759/cureus.20048

**Published:** 2021-11-30

**Authors:** Peter Drevets, Hossam Alslaim, Irfan Saeed

**Affiliations:** 1 General Surgery, Augusta University Medical College of Georgia, Augusta, USA; 2 Division of Transplant Surgery, Augusta University Medical College of Georgia, Augusta, USA

**Keywords:** novel transplantation techniques, renal transplant revision, third renal transplant, renal transplantation, prune belly syndrome

## Abstract

Kidneys are the most frequently transplanted organ in the United States. An infrequently encountered cause of end-stage renal disease requiring permanent dialysis is prune belly syndrome. Prune belly syndrome is mostly seen in males and over a third of patients require permanent dialysis. Due to defects in abdominal wall musculature, transplantation in these patients is technically challenging. We present a novel case of a third renal transplant in a patient with prune belly syndrome with two previous failed transplants.

## Introduction

Kidneys are the most transplanted organs in the United States. In 2020, there were 39,000 renal transplants performed [1]. Rapid advances in immunosuppressive therapy and transplant medicine have been made renal transplantation a viable option for patients with end-stage renal disease (ESRD). Patients with prune belly syndrome (PBS) are frequently associated with ESRD and renal transplantation. Prune belly syndrome (PBS) is most characterized by the lack of abdominal wall musculature, genitourinary defects, and cryptorchidism [2]. Ninety-seven percent of patients with PBS are male and 30% will become candidates for kidney transplantation [2]. The etiology of the syndrome remains unknown but multiple theories have been proposed. One of the most accepted theories refers to urethral obstruction-malformation complex resulting in bladder distension with secondary effects on the development of the abdominal wall, urinary tract, and testicular descent. Patients with PBS have a transplant survival graft rate similar to transplant recipients without the condition [3]. We present an exceptionally rare case of a patient with PBS who had undergone two previous renal transplants, which both subsequently failed. We performed a third renal transplant at our institution. To our knowledge and based on our examination of the literature, a third renal transplant in a patient with prune belly syndrome has not been previously described. Therefore, we believe our report of the procedure will serve as a rare description that a third renal transplant can be performed in patients with this condition.

## Case presentation

A 33-year-old African-American male with prune belly syndrome presented to our center for renal transplant evaluation after two previous failed transplants. The patient received his first transplant from a living donor at age 10. The patient’s right native kidney was removed at the time of the first transplant and the transplanted kidney was placed in the right lower quadrant. The kidney functioned for 12 years before failing due to chronic allograft dysfunction. He then received dialysis for three years before a second renal transplant from a cadaveric donor was performed. The initial transplant kidney in the right lower quadrant was removed and the deceased donor renal transplant kidney was placed in the left lower quadrant. The second transplant kidney lasted for six years before failing due to lack of compliance with immunosuppressive medication.

At the time of evaluation, the patient was receiving dialysis thrice weekly via an upper-extremity arterio-venous fistula and was producing a miniscule amount of urine per day. A computed tomography scan performed at the time of evaluation demonstrated an atrophic left native kidney, an empty right renal fossa with surgical clips, and a renal transplant in the left lower quadrant. After appropriate workup and counseling, the patient was placed on the transplant recipient list. Once a compatible kidney became available, the patient was called in and final histocompatibility labs were performed and found to be non-reactive. 

Prior to the patient arriving in the operating room, the donor kidney was prepared on the back table in the operating theater. Due to the patient's history of prior renal transplants and need for intra-abdominal allograft implantation, a cavoplasty was not performed. 

The abdomen was accessed using a lower midline incision and noted the expected lack of abdominal musculature typical of PBS. The dissection was uneventful and the inferior vena cava (IVC) and right common iliac artery were exposed in preparation for anastomosis of the donor organ. The inferior vena cava (IVC) was clamped and venotomy created to accommodate donor renal vein. We then performed an end-to-side anastomosis of the donor renal vein and the inferior vena cava. The transplant renal artery was then anastomosed to the right common iliac artery in an end-to-side fashion. Good renal perfusion was noted immediately after unclamping. Implantation of the ureter into the bladder was performed with double-j ureteral stents and the bladder tacked to the anterior abdominal wall. Immediate urine production was noted in the recovery unit.

The patient received routine post-operative care for renal transplantation and was discharged on a post-operative day four with an immunosuppressive regimen consisting of extended-release tacrolimus, mycophenolate mofetil, and prednisone. Ureteral stents were removed one month following the procedure. At the time of writing this report, the patient had been seen in the transplantation clinic routinely and has been adherent to transplant immunosuppressive medications and is progressing well.

## Discussion

Third renal transplants are technically challenging from a surgical standpoint. However, survival remains improved when compared to continued dialysis [4]. Thus, we strongly believe that transplantation must be considered for all ESRD patients, even if potentially complicated. Although the procedure may be more complicated, the potential benefit to life expectancy remains significant. The importance of adequate follow-up and clinical support cannot be overemphasized following a transplant. The surgery is just one step in the lifelong process required for successful transplantation. Our center utilizes a multidisciplinary team consisting of physicians, nurses, and pharmacists to provide comprehensive pre- and post-transplant care. We believe this allows for a holistic and all-encompassing approach to individualize transplant-related care. As seen in our patient prior to the procedure, non-compliance can have significant negative results. Education and support provided by a multidisciplinary team are as important as an experienced surgeon. Non-compliance is likely a multifactorial issue, but with proper education and support, the incidence can be minimized.

## Conclusions

PBS is just one of many conditions leading to ESRD that can be effectively managed with transplantation. With continued improvement in transplant transport techniques and immunosuppressive regimens, renal transplantation will continue to be an important life-saving therapy for patients. In spite of modern medical advances, some patients will require repeat transplantation procedures, such as our patient. We believe that the benefits are significant and repeat surgery, although potentially complex, should be considered. Patients with PBS present on a spectrum of varying pathologies and complexities rendering renal transplant challenging in a subgroup of those patients. The lack of abdominal wall musculature and congenital urogenital tract defects potentially increases the risk of surgical complications and might affect kidney transplant longevity. Despite those challenges, we believe this unique report will serve as a record stating third renal transplantation is a viable option for PBS patients with prior donor-organ failure.
